# Prevalence of *Balantidium coli* (Malmsten, 1857) infection in swine reared in South Italy: A widespread neglected zoonosis

**DOI:** 10.14202/vetworld.2021.1044-1049

**Published:** 2021-04-30

**Authors:** Filippo Giarratana, Luca Nalbone, Ettore Napoli, Vincenzo Lanzo, Antonio Panebianco

**Affiliations:** 1 Department of Veterinary Science, University of Messina, Polo Universitario dell’Annunziata, 98168 Messina, Italy; 2 Freelance Veterinary Professional , 89024 Polistena (RC), Italy

**Keywords:** *Balantidium coli*, ciliate protozoa, protozoa, swine, zoonosis

## Abstract

**Background and Aim:**

Balantidiasis, caused by *Balantidium coli* (syn. *Neobalantidium coli* or *B. coli*), represents a neglected parasitic infection of zoonotic significance affecting a variety of hosts, including domestic pigs that are the main reservoir. *B. coli* has a direct life cycle with a fecal-oral route transmission that occurs mainly by the ingestion of food and water contaminated with cysts. The ingestion of meat contaminated during inappropriate slaughtering processes may represent a new potential route of transmission. Only a few studies have investigated the prevalence of *B. coli* in domestic pigs in Italy, despite its high prevalence and zoonotic significance. This study aimed to improve the knowledge on *B. col*i prevalence in domestic swine reared both in intensive and “en plein air” breeding systems in the south of Italy.

**Materials and Methods:**

The infection rate of *B. coli* in pigs bred in 15 different pig farms and regularly slaughtered in South Italy, in the Calabria region, was investigated. From 2017 to 2019, 177 terminal parts of the rectums of pigs, of which 91 commercial hybrids and 86 autochthonous (Nero Calabrese), reared, respectively, in intensive and “en plein air” breeding system, were tested for parasite detection. After the slaughtering, the terminal part of the rectum was sampled and transported to the laboratories and immediately processed. For the detection of trophozoites, fresh smears of feces were examined at light microscopy. Moreover, flotation was performed using a sodium chloride solution, then smears of feces were dispersed on microscope slides and examined at light microscopy. The observed parasites were identified at the species level using morphological characteristics.

**Results:**

*B. coli* was detected in a total of 83 (i.e., 46.89%) pigs, with a significantly higher prevalence (p<0.0001) found in commercial hybrid (i.e., 59/91-64.84%) pigs rather than in autochthonous ones (i.e., 24/86-27.91%). It follows that the infection was more common in pigs reared in the intensive breeding system than in “en plein air” ones (p<0.0001). The infection prevalence was higher in males than in females and lower in elder animals than in younger ones (p=0.012 and p<0.0001, respectively).

**Conclusion:**

The breeding system is likely the main discriminant for the parasite spread as well as the hygienic condition of the farms. Good manufacturing and hygiene practices along the food production chain are crucial in preventing human balantidiasis transmission by meat consumption. The high *B. coli* prevalence, the wide spectrum of host species, and its zoonotic significance push toward a greater public interest.

## Introduction

Basic health surveillance of livestock farms aims to contrast the development and the spread of infectious disease which may lead to significant economic losses and could be transmitted to humans [1,2]. Constant monitoring plans are essential since the diffusion of certain hazards in a new geographical area may develop due to changes in trade patterns, breeding models, environment, climate, and social life [3]. Balantidiasis, caused by *Balantidium coli* (syn. *Neobalantidium coli* or *B. coli*), represents a neglected parasitic infection of zoonotic significance affecting a variety of hosts including human [4-6]. *B. coli* (Malmsten, 1857), a ciliated protozoan, belonging to the family Balantidiidae [7], is considered a commensal of the intestine of several mammalian hosts (i.e., pig, human, camel, monkey, and rarely dog and rat) [8,9]. The reservoir host is the domestic and wild pigs, in which the parasite inhabits mainly the *villi* or *lumen* of the large intestine [10,11]. In the life cycle of the parasite, there are two stages, cyst and trophozoite. The cyst is the non-replicating form, excreted with feces, inside of could survive for weeks [12], and is responsible for the infection of new hosts. The trophozoite is vegetative form that normally is detected in the host intestine and could survive only a few hours outside the host [8,13]. *B. coli* has a direct life cycle with a fecal-oral route transmission that occurs mainly by the ingestion of food and water contaminated with cysts [14,15]. It has a worldwide distribution, predominantly in the subtropical and tropical regions of the world [16]. Higher temperature and humidity favor the development and survival of this parasite [13,17].

In developing countries, balantidiasis could be a serious threat and the spread of the disease is related to the contamination of water and food sources with swine feces [14,15]. Poor hygienic conditions, the coexistence of humans and animals, malnutrition, concomitant infections, and debilitating diseases are all predisposing factors for the development and spread of balantidiasis among animals and humans [18]. In developed countries, as a waterborne parasite, *B. coli* could be transmitted by recreational water (i.e., in swimming pools) and their occasional contamination or as process failure within water utilities [19]. In the definitive host, *B. coli* is commonly considered a non-pathogenic parasite; however in symptomatic cases, the clinical presentation is characterized by loose feces, fetid watery diarrhea, appetite loss, dehydration, loss of body condition, and retarded growth, inevitably leading to economic loses [20,21]. In some cases, even in human *B. coli* infection could cause a severe syndrome, characterized by mucosal ulceration, accompanied by diarrhea, and dysentery with possible fatal outcomes [22]. In domestic pigs, the worldwide *B. coli* prevalence reported ranges from 0.7 to 100% [9,23-30]. However, given the apparent low pathogenicity in animals, balantidiasis was not considered a disease of relevant public interest. Nevertheless, their high incidence worldwide, the possible fatal outcome in humans, the continuous change in the livestock sector, and the evolution of international markets, stress the importance of not underestimating balantidiasis in both developing and developed countries [8,31-33].

Over the past decade, few studies have investigated the prevalence of *B. coli* in domestic pigs and, to the authors’ best knowledge, only a previous report has been carried out in Italy [9]. This study aimed to improv the knowledge on *B. col*i prevalence in domestic swine reared both in intensive and “en plein air” breeding systems, regularly slaughtered in South Italy.

## Materials and Methods

### Ethical approval

Ethical approval was not necessary for this study because study was conducted on dead animals.

### Study period and location

In the present study, we have investigated the occurrence of *B. coli* in the south of Italy, in Calabria region, comparing its prevalence between commercial hybrid and autochthonous pigs reared in intensive and “en plein air” breeding systems, respectively (Table-1). Sampling and sample processing were carried out from June 2017 to February 2019. The sample processing was performed at the Food Inspection Laboratory of the Department of Veterinary Sciences at the University of Messina, Italy.

**Table 1:** Distribution of the study populations and positive samples with relative prevalence of *Balantidium coli.*

Breeding system	No. of samples	No. of positive samples (%)	Breed				Gender				Age in months			
		
			Commercial hybrid		Autochthonous		Male		Female		<10		>10	
					
			Total	Positive (%)	Total	Positive (%)	Total	Positive (%)	Total	Positive (%)	Total	Positive (%)	Total	Positive (%)
Intensive	91	59 (64.84)	91	59 (64.84)	0	0	46	39 (84.78)	45	20 (44.44)	91	59 (64.84)	0	0
En plein air	86	24 (27.91)	0	0	86	24 (27.91)	44	12 (13.94)	42	12 (13.94)	0	0	86	24 (27.91)
Total	177	83 (46.89)	91	59 (64.84)	86	24 (27.91)	90	51 (56.67)	87	32 (36.78)	91	59 (64.84)	86	24 (27.91)

### Study population and sampling

The study population consisted of commercial hybrid pigs obtained by the crossbreeding of Pietrain x Large White and autochthonous breed pigs called Nero Calabrese. The commercial hybrid was lightweight pigs (i.e., 90-120 kg) breed mainly for fresh meat production. Nero Calabrese is an autochthonous black-haired breed. The breeding of Nero Calabrese has ancient origins, and in fact, written documents testify the presence of this breed since the period of Greek and Carthaginian domination. Today, the breading of the autochthonous breeds is gaining importance not only for their preservation but also for the quality of the meat and the fat and the greater rusticity and adaptation to the local environment of these swine species. Pigs breed for food purpose, at the end of the breeding cycle, were brought to the slaughterhouse. For each animal, an individual sheet registering data on farm origin, gender, breed, and age were filled. The prevalence of *B.coli* was investigated in a total of 177 pigs (i.e., 90 males and 87 females) from 15 different pig farms. In particular, 91 (i.e., 46 males and 45 females) were commercial hybrids aged from 7 to 8 months and 86 were Nero Calabrese (i.e., 44 males and 42 females) elder than 10 months. All the commercial hybrids were bred in intensive system in eight different pig farms while all the autochthones ones were breed in “en plein air” system in seven different pig farms.

### Laboratory procedures

After the slaughtering, the terminal part of the rectum was sampled and transported at the controlled temperature of 39±1°C to the laboratories of the Department of Veterinary Sciences (University of Messina) for the parasitological analysis and immediately processed. For the detection of trophozoites, fresh smears of feces suspended in preheated 0.9% (w/v) NaCl were dispersed on microscope slides and examined at light microscopy (DM2000, Leica, Germany; 400-1000×), according to Giarratana *et al*. [9]. From each animal, an additional fecal sample (i.e., about 5 g) was individually collected and processed by a flotation method using saturated preheated NaCl solution (1200 Newton/m^3^), according to the method proposed by Wade and Gaafar [34], to confirm the protozoa presence. A pig was classified as positive if at least one cyst or trophozoite was found in at least one of the methods used. The observed parasites were identified at the species level using the morphological characteristics [35].

### Statistical analysis

The frequency of *B. coli* infection in each animal farm/category was calculated by dividing the total of the positive samples by the total samples collected and expressed as a percentage. Data analysis was performed using statistical software program Prism v. 8.00 (GraphPad Software Ltd., CA, USA). To assess any potential differences in *B. col*i prevalence between variables including age, gender, breed, and breeding system, the ^χ2^ test or unpaired t-tests were carried out as appropriate (with 95% confidence level) [36].

## Results

The information on the study population is summarized in Table-1. *B. coli* out of 177 samples analyzed, 83 (i.e., 46.89%) tested positive at least to one of the two methods used for parasite detection. The infection rate was significantly (p*<*0.0001) higher in commercial hybrid (i.e., 59/91-64.84%) rather than in the autochthonous breed (i.e., 24/86-27.91%). The parasite prevalence was significantly (p<0.0001) higher in pigs reared in intensive system than in “en plein air” ones. Significant differences in *B. coli* prevalence were observed between gender (p*=*0.012) and age (p<0.0001). In fact, the infection was more common in males (i.e., 51/90, 56.67%) than in females (i.e., 32/87, 36.78%); the prevalence observed in elder animals (i.e., 24/86, 27.91%) was significantly lower compared to the younger ones (i.e., 59/91, 64.84%).

## Discussion

The data gained in the present study underline that *B. coli* is a neglected but widespread parasitosis; in fact, almost half of the pigs tested were positive. The findings herein reported do not differ from national and European situations. In this regard, although the data on *B. coli* prevalence are mostly dated and gained on small sample size population, the presence of this parasite has been described with a prevalence ranging from 31.3% to 100% [29,30,37-42]; suggesting that, probably due to the low pathological importance, this parasitosis has been poorly considered despite its zoonotic relevance. The obtained results hint that the breeding system and its management are discriminant factors in *B. coli* prevalence and spread. Nowadays, the enhancement of more sustainable breeding systems (i.e., outdoor system, “en plein-air” farming, etc.), the autochthonous breeds rediscovery and the need for specific food chains risk assessment, stress the importance of understanding the differences between different breeding systems. We found a significantly higher infection rate in commercial hybrid breed in intensive system rather than in autochthonous ones bred in “en plein air” system. These results are consistent with the data obtained in a previous study conducted in Sicily, South Italy [9]. In the former, a higher parasite prevalence has been found in commercial hybrid pigs (i.e., 106/123-86.18%) rather than in autochthonous ones (Nero Siciliano) (i.e., 44/134-32.84%) reporting even greater infection rate. In this regard, using the same analytical method, the overall prevalence of *B. coli* (58.37%) was higher than that herein reported. On the other hand, other authors have reported that animals reared with tie stalls management are less prone to the infection than those reared on free stall management system where the hygienic and antiparasitic control plans are harder to manage. The higher prevalence herein reported, the higher prevalence in commercial hybrid is probably related to the maintenance systems and the hygienic condition of the farms that are relevant risk factors for parasite spread [8,12]. In this regard, Sangioni *et al*. [43] reported the presence of *B. coli* in several swine farms kept in intensive farming systems stressing that swine farms with better hygienic and sanitary standards had lower parasitic infection rate. Inadequate farm management [44] could represent a serious threat since, being the animals in close contact, the spread of *B. coli* is highly enhanced. This last finding is corroborated by the fact that in young animals maintained in close contact [29], *B. coli* spread is higher than in adult animals, as also herein observed. Considering the results obtained both in the present and in a previous study [9], it seems that the autochthonous breeds (i.e., Nero Calabrese and Nero Siciliano) are more resistant to *B. coli* infection compared to the commercial hybrid. However, this finding could also be related to breeding technique. The “en plein air” system could play a key role in reducing the infection spread determining a “dilution” effect on protozoa excreted with feces, since pigs, having more space available, are less in contact with each other. The type of diet contributing to the breed’s rusticity could be a relevant parameter that could generate a resistance to the infection. Therefore, further studies are needed to increase the knowledge of the effective refractory of these breeds to *B. coli* infection. According to other previous studies [45,46], the data herein presented allow to speculate that gender could be considered a risk factor since the infection was more common in males than in females. However, a shared consensus does not exist: Some authors report a higher prevalence in males [47], conversely others observed a higher one in females [48]. In intensive breeding system, the increasing use of artificial fecundation reduces the contact among animals; furthermore, the boars are normally maintained in separated pen to avoid aggression related to dominance [49], while the sows are usually housed in piggeries, in close contact with each other, then more exposed to the infection. In contrast, a higher number of antiparasitic treatment were performed to the sows, to increase the reproductive performances and to ameliorate the wellness of the suckling piglets. Noteworthy, any of the pigs sampled in the present study show clinical symptoms; therefore, the widespread of *B. coli* related to asymptomatic carriers raises concerns for public health.

As waterborne and foodborne zoonosis, balantidiasis may lead to a serious infection in human as severe diarrhea and other gastrointestinal disorders [50]; noteworthy, recently, *B. coli* infection in humans was associated to serious diseases, such as liver abscess that mimicking a tumor [51] or to vertebral osteomyelitis and myelopathy [52]. Prevention of water and food contamination represents the best way to avoid the disease spread. The high prevalence of protozoa in the feces of slaughtered pigs needs attention since carcasses contamination during slaughtering could be considered as a risk factor for hygiene and safety of meat production. In fact, while contamination of water sources with human or swine faces is the main pathway of transmission in developing countries [14], the consumption of raw or undercooked meat could represent an important route of human infection in developed countries. *B. coli* inhabits in the last intestinal tracts of animals; therefore, meat contamination could occur during the evisceration step in slaughterhouses; moreover, inadequate hygiene practices could lead to cross-contamination among the carcasses [9]. In this regard, Panebianco [53] detected *B. coli* cysts on surface scraping samples of swine carcasses. Furthermore, protozoan transmission from feces to meat poses concerns also for meat preparation. In this respect, Schuster and Visvesvara [54] observed that, in favorable conditions, *B. coli* can survive for weeks in porcine faces; therefore, its persistence on carcasses surface along the production line may lead to contamination of this kind of foodstuffs. In this regard, compliance with the specific hygiene requirements for slaughterhouse demanded by Regulation (EC) 853/04 [55] and relative official controls according to Regulation (EC) 625/2017 [56] and Regulation (EC) 627/2019 [57] ensure proper risk management of the fecal contamination and cross-contamination of carcasses. Furthermore, the constant monitoring of the microbiological process hygiene criteria according to Regulation (EC) 2073/05 [58] allows effective control of fecal contamination levels of carcasses [59,60]. This monitoring can be considered effective also against fecal parasite contaminations. The risk management is carried out already starting from the documentary check of the Food Chain Information (FCI) contained in the records of the primary producers according to Regulation (EC) 852/2004 [61]. Based on Regulation (EC) 627/2019 [57], the official veterinarian communicates relevant results of antemortem and postmortem inspections to the primary producers including any insufficient cleaning status of the animals. In this way, according to a risk-based approach, higher attention will be paid to those batch of animals coming from holdings whom FCI report relevant food safety information.

## Conclusion

The high *B. coli* prevalence reported in this study reflects the importance of not underestimating this parasitosis. Despite the high *B. coli* prevalence, the wide spectrum of host species (i.e., from pigs to non-human primates [35]), and its zoonotic significance, to date, there is a lack of knowledge on its worldwide prevalence. Considering the ability of *B. coli* to potentially survive on carcasses surface of slaughtered animals [53], good manufacturing and hygiene practices play the most important role in preventing its spread. However, further studies on the survival of *B. coli* cyst in the environment and on food matrices should be carried out, as well as the evaluation of other potential human infections sources is needed.

## Authors’ Contributions

FG and AP conceived the study. FG and VL performed the experiment. FG, LN, and EN analyzed the data. FG, LN, and EN drafted and revised the manuscript. All authors read and approved the final manuscript.

## References

[1] Yazdanbakhsh O, Zhou Y, Dick S (2017). An intelligent system for livestock disease surveillance. Inf. Sci.

[2] Bauerfeind R, Bauerfeind R, Von Graevenitz A, Kimmig P, Schiefer H.G, Schwarz T, Slenczka W, Zahner H (2016). Zoonoses:Infectious Diseases Transmissible from Animals to Humans.

[3] Bloom D.E, Black S, Rappuoli R (2017). Emerging infectious diseases:A proactive approach. PNAS.

[4] Areán V.M, Koppisch E (1956). Balantidiasis:A review and report of cases. Am. J. Pathol.

[5] Cho H.S, Shin S.S, Park N.Y (2006). Balantidiasis in the gastric lymph nodes of Barbary sheep (*Ammotragus lervia*):An incidental finding. J. Vet. Sci.

[6] Paul T.R, Begum N, Shahiduzzaman M, Hossain M.S, Labony S.S, Dey A.R (2019). Balantidiasis, a zoonotic protozoan infection, in cattle and domestic pigs. Bangladesh J. Vet. Med.

[7] Lee J.J, Hutner S, Bovee E, Lee J.J, Leedale G.F, Bradbury P (2000). An Illustrated Guide to the Protozoa:Organisms Traditionally Referred to as Protozoa, or Newly Discovered Groups. Society of Protozoologists.

[8] Ahmed A, Ijaz M, Ayyub R.M, Ghaffar A, Ghauri H.N, Aziz M.U, Ali S, Altaf M, Awais M, Naveed M, Nawab Y, Javed M.U (2020). *Balantidium coli* in domestic animals:An emerging protozoan pathogen of zoonotic significance. Acta Tróp.

[9] Giarratana F, Muscolino D, Taviano G, Ziino G (2012). *Balantidium coli* in pigs regularly slaughtered at abattoirs of the province of Messina:Hygienic observations. Open J. Vet. Med.

[10] Soulsby E.J.L (1982). Helminths, Arthropods and Protozoa of Domesticated Animals.

[11] Jones T.C, Hunt R.D, King N.W (1997). Veterinary Pathology.

[12] Wisesa I.B.G, Siswanto F.M, Putra T.A, Oka I.B.M, Suratma N.A (2015). Prevalence of *Balantidium* sp in Bali cattle at different areas of Bali. Int. J. Agric. For. Plant.

[13] Ponce-Gordo F, García-Rodríguez J.J (2020). Balantioides coli. Res. Vet. Sci.

[14] Plutzer J, Karanis P (2016). Neglected waterborne parasitic protozoa and their detection in water. Water Res.

[15] Thompson R.C.A, Smith A (2011). Zoonotic enteric protozoa. Vet. Parasitol.

[16] Shabih H.S, Juyal P.D (2006). Epidemiological Observations of Paramphistomosis in Ruminants in Endemic Regions of Punjab and Adjoining State. In:Proceedings of the 11^th^ International Symposium on Veterinary Epidemiology and Economic, India.

[17] Datta S, Chowdhury M.K, Siddiqui M.A.R, Karim M.J (2004). A retrospective study on the prevalence of parasitic infection in ruminants in selected areas of Bangladesh. Bangladesh J. Vet. Med.

[18] Solaymani-Mohammadi S, Petri W.A (2006). Jr. Zoonotic implications of the swine-transmitted protozoal infections. Vet. Parasitol.

[19] Bellanger A.P, Scherer E, Cazorla A, Grenouillet F (2013). Dysenteric syndrome due to *Balantidium coli*:A case report. New Microbiol.

[20] Palanivel K.M, Thangathurai R, Nedunchellian S (2005). Epizootiology of *Balantidium coli* infection in ruminants. Indian Vet. J.

[21] Roy B.C, Mondal M.M.H, Talukder M.H, Majumder S (2011). Prevalence of *Balantidium coli* in Buffaloes at different areas of Mymensingh. J. Bangladesh. Agric. Univ.

[22] Yazar S, Altuntas F, Sahin I, Atambay M (2004). Dysentery caused by *Balantidium coli* in a patient with non-Hodgkin's lymphoma from Turkey. World J. Gastroenterol.

[23] Yin D.M, Lv C.C, Tan L, Zhang T.N, Yang C.Z, Liu Y, Liu W (2015). Prevalence of *Balantidium coli* infection in sows in Hunan province, subtropical China. Trop. Anim. Health Prod.

[24] Ismail H.A.H, Jeon H.K, Yu Y.M, Do C, Lee Y.H (2010). Intestinal parasite infections in pigs and beef cattle in rural areas of Chungcheongnam-do, Korea. Korean J. Parasitol.

[25] Schuster F.L, Ramirez-Avila L (2008). Current world status of *Balantidium coli*. Clin. Microbiol. Rev.

[26] Yatswako S, Faleke O.O, Gulumbe M.L, Daneji A (2007). I. *Cryptosporidium* oocysts and *Balantidium coli* cysts in pigs reared semi-intensively in Zuru, Nigeria. Pak. J. Biol. Sci.

[27] Weng Y.B, Hu Y.J, Li Y, Li B.S, Lin R.Q, Xie D.H, Grasser R.B, Zhu X.Q (2005). Survey of intestinal parasites in pigs from intensive farms in Guangdong Province, People's Republic of China. Vet. Parasitol.

[28] Wieler L.H, Ilieff A, Herbst W, Bauer C, Vieler E, Bauerfeind R, Failing K, Klös H, Wengert D, Baljer G, Zahner H (2001). Prevalence of enteropathogens in suckling and weaned piglets with diarrhoea in Southern Germany. J. Vet. Med. B Infect. Dis. Vet. Public Health.

[29] Hindsbo O, Nielsen C.V, Andreassen J, Willingham A.L, Bendixen M, Nielsen M.A, Nielsen N.O (2000). Age-dependent occurrence of the intestinal ciliate *Balantidium coli* in pigs at a Danish research farm. Acta Vet. Scand.

[30] Quilez J, Sánchez-Acedo C, Clavel A, Del Cacho E, Lopez-Bernad F (1996). Prevalence of *Cryptosporidium* infections in pigs in Aragon (Northeastern Spain). Vet. Parasitol.

[31] Fletcher S.M, Stark D, Harkness J, Ellis J (2012). Enteric protozoa in the developed world:A public health perspective. Clin. Microbiol. Rev.

[32] Kline K, McCarthy J.S, Pearson M, Loukas A, Hotez P.J (2013). Neglected tropical diseases of Oceania:Review of their prevalence, distribution, and opportunities for control. PLoS Negl. Trop. Dis.

[33] Rojas-Downing M.M, Nejadhashemi A.P, Harrigan T, Woznicki S.A (2017). Climate change and livestock:Impacts, adaptation, and mitigation. Clim. Risk Manag.

[34] Wade W.F, Gaafar S.M, Colville J (1991). Common laboratory procedures for diagnosing parasitism. Diagnostic Parasitology for Veterinary Technicians.

[35] Ponce-Gordo F, Jimenez-Ruiz E, Martínez-Díaz R.A (2008). Tentative identification of the species of *Balantidium* from ostriches (*Struthio camelus*) as *Balantidium coli*-like by analysis of polymorphic DNA. Vet. Parasitol.

[36] Dytham C (1999). Choosing and Using Statistics:A Biologist's Guide. Blackwell Science.

[37] De Carneri I (1959). Nuove osservazioni su *Balantidium coli*. Diffusione tra i suini a milano, coltivazione, sensibilitàai farmaci *in vitro*. Riv. Parassitol.

[38] Favati V (1959). Sulla Balantidiosi Suina in Toscana. Ann. Facolt. Med. Vet. Pisa.

[39] Nardi E (1960). La Balantidiosi nei Suini e nei Bovini del Comune di Foggia. Vet. Ital.

[40] Iannuzzi L (1967). Indagini sulla Frequenza di *Balantidium col*i (Malmsten, 1857) Negli Animali da Macello. Ann. Facol. Med. Vet. Messina.

[41] Eydal M, Konráösson K (1998). The prevalence of *Balantidium coli* and other zoonotic parasites in Icelandic pigs. Parasitol. Int.

[42] Polinas L, Cocco G, Tanda B, Basciu M, Coccone G.N.S, Marrosu R, Pipia A.P, Nieddu M.S, Scala A (2006). Gastro-intestinal parasites of pigs in Sardinia:A copromicroscopical investigation. Parassitologia.

[43] Sangioni L.A, de Avila Botton S, Ramos F, Cadore G.C, Monteiro S.G, Pereira D.I. B, Vogel F.S.F (2017). *Balantidium coli* in pigs of distinct animal husbandry categories and different hygienic-sanitary standards in the central region of Rio Grande do Sul state, Brazil. Acta Sci. Vet.

[44] Mundim M.J.S, Mundim A.V, Santos A.L.Q, Cabral D.D, Faria E.S.M, Moraes F.M (2004). Helmintos e protozoários em fezes de javalis *(Sus scrofa scrof*a) criados em cativeiro. Arq. Bras. Med. Vet. Zootec.

[45] Hussin A.G, Al-Samarai F.R (2016). Prevalence of *Balantidium coli* in cattle and cattle breeders in some regions of Baghdad in Iraq. Bangladesh J. Vet. Med.

[46] Yaghoobi K, Sarkari B, Mansouri M, Motazedian M.H (2016). Zoonotic intestinal protozoan of the wild boars, *Sus scrofa*, in Persian Gulf's coastal area (Bushehr Province), Southwestern Iran. Vet. World.

[47] Abdullah-Al-Hasan M, Rahman M.A, Saha B.K, Abdullah-Al-Hasan A.T.M, Rakib A.F.K, Mondal M.H (2015). Occurrence of *Balantidium coli* in pig in Mymensingh, Bangladesh. Int. J. Nat. Soc. Sci.

[48] Bilal C.Q, Khan M.S, Avais M, Ijaz M, Khan J.A (2009). Prevalence and chemotherapy of *Balantidium coli* in cattle in the River Ravi region, Lahore (Pakistan). Vet. Parasitol.

[49] Kanis E, De Greef K.H, Hiemstra A, Van Arendonk J.A.M (2005). Breeding for societally important traits in pigs. J. Anim. Sci.

[50] McLeod C, Smith P, McGuinness S.L, Francis J.R, Baird R.W (2015). Human case of *Balantidium* infection in Australia. Pathology.

[51] Kapur P, Das A.K, Kapur P.R, Dudeja M (2016). *Balantidium coli* liver abscess:First case report from India. J. Parasit. Dis.

[52] Dhawan S, Jain D, Mehta V (2013). S. *Balantidium coli*:An unrecognized cause of vertebral osteomyelitis and myelopathy:Case report. J. Neurosurg.

[53] Panebianco F (1967). Igiene delle Carni e *Balantidium col*i (Malmsten, 1857). Ann. Facolt. Med. Vet. Messina.

[54] Schuster F.L, Visvesvara G.S (2004). Amebae and ciliated protozoa as causal agents of waterborne zoonotic disease. Vet. Parasitol.

[55] The European Parliament and the Council of the European Union. Regulation (EC) 853/04 of the European Parliament and of the Council of 29 April 2004:Laying down Specific Hygiene Rules for on the Hygiene of Foodstuffs (2004). Official Journal of the European Union, No. L139/55.

[56] The European Parliament and the Council of the European Union Regulation (EC) 625/2017 of the European Parliament and of the Council of 15 March 2017:On Official Controls and other Official Activities Performed to Ensure the Application of Food and Feed Law, Rules on Animal Health and Welfare, Plant Health and Plant Protection Products (Official Controls Regulation), Official Journal of the European Union. (2017) No. L95/1.

[57] The European Parliament and the Council of the European Union Regulation (EC) 627/2019 of the European Parliament and of the Council of 15 March 2019:Laying down Uniform Practical Arrangements for the Performance of Official Controls on Products of Animal Origin Intended for Human Consumption in Accordance with Regulation (EU) 2017/625 of the European Parliament and of the Council and amending Commission Regulation (EC) No 2074/2005 as regards official controls, Official Journal of the European Union, No. (2019) L131/51.

[58] The European Parliament and the Council of the European Union Regulation (EC) 2073/2005 of the European Parliament and of the Council of 15 November 2005:On Microbiological Criteria for Foodstuffs, Official Journal of the European Union, No. (2005) L338/1.

[59] Giuffrida A, Ziino G, Panebianco A, Cassarà S (2003). Inspective consideration in detection of enterobacteria in the blood of normally slaughtered swine. Vet. Res. Commun.

[60] Napoli E, Nalbone L, Giarratana F (2021). Balantidiasis a potential neglected zoonotic disease and the liar paradox. Biosci. Biotechnol. Res. Asia.

[61] The European Parliament and the Council of the European Union Regulation (EC) 852/04 of the European Parliament and of the Council of 29 April 2004:On the Hygiene of Foodstuffs, Official Journal of the European Union, No. (2004) L139/1.

